# Combination Therapy of Bland Transarterial Embolization and Microwave Ablation for Hepatocellular Carcinoma within the Milan Criteria Leads to Significantly Higher Overall Survival

**DOI:** 10.3390/cancers15205076

**Published:** 2023-10-20

**Authors:** Hamzah Adwan, Moath Adwan, Thomas J. Vogl

**Affiliations:** Department of Diagnostic and Interventional Radiology, University Hospital, Goethe University Frankfurt, Theodor-Stern-Kai 7, 60590 Frankfurt, Germany

**Keywords:** hepatocellular carcinoma, Milan criteria, microwave ablation, bland transarterial embolization

## Abstract

**Simple Summary:**

Image-guided interventional treatments play an important role in treating HCC. Percutaneous thermal ablation is suitable for early-stage HCC, and transarterial therapies are recommended for intermediate-stage HCC. Several studies evaluated the combination therapy of TACE and RFA, in comparison to monotherapy of RFA or TACE alone, but there is still a lack of studies that investigated the combination therapy of bland embolization (without the application of chemotherapeutics) with thermal ablation. This study aims to compare the combination therapy of lipiodol-based TAE and MWA with MWA alone for primary HCC.

**Abstract:**

A comparison of the combination therapy consisting of microwave ablation (MWA) after bland lipiodol-based transarterial embolization (TAE) with MWA alone in the treatment of hepatocellular carcinoma (HCC) within the Milan criteria. Forty-nine patients in the TAE-MWA group (12 women and 37 men; mean age: 63.3 ± 9.6 years) with 55 tumors and 63 patients in the MWA group (18 women and 45 men; mean age: 65.9 ± 10.5 years) with 67 tumors were retrospectively enrolled in this study. For the investigation of treatment protocols based upon both safety and efficacy, patients’ cases were analyzed with regard to complications, local tumor progression (LTP), intrahepatic distant recurrence (IDR), overall survival (OS), and progression-free survival (PFS). There were no cases of major complications in either group. The LTP rate was 5.5% in the MWA-TAE group and 7.5% in the MWA group (*p* = 0.73). The rate of IDR was 42.9% in the MWA-TAE group and 52.4% in the MWA group (*p* = 0.42). The 12-, 24-, and 36-month OS rates starting at the date of tumor diagnosis were 97.7%, 85.1%, and 78.8% in the TAE-MWA group, and 91.9%, 71.4%, and 59.8% in the MWA group, respectively (*p* = 0.004). The 6-, 12-, and 24-month PFS rates were 76.5%, 55%, and 44.6% in the TAE-MWA group, and 74.6%, 49.2%, and 29.6% in the MWA group, respectively (*p* = 0.18). The combination therapy of TAE-MWA was significantly superior to MWA monotherapy according to OS in treating HCC within the Milan criteria.

## 1. Introduction

Image-guided, minimally invasive interventional treatments play an essential role in treating hepatocellular carcinoma (HCC).

According to the European Association for the Study of Liver Disease (EASL), local thermoablative therapies are recommended for patients with very early/early disease (BCLC 0 and A) and transarterial chemoembolization (TACE) as a first-line treatment option for patients with intermediate HCC (BCLC B) [1]. 

Hyperthymia ablation therapies for HCC include radiofrequency ablation (RFA) and microwave ablation (MWA) [1]. MWA is less affected by the heat sink effect, offers larger ablation areas, and is faster than RFA [2]. MWA and RFA have comparable efficacy; however, MWA is especially superior when treating larger tumors [3].

While TACE is performed by injecting chemotherapeutics followed by embolization materials into the tumor-feeding artery, bland transarterial embolization (TAE) is carried out by solely injecting embolization materials into the tumor-supplying artery [4].

Whether TACE should be favored over TAE in treating HCC remains controversial, for TACE’s significant superiority in terms of overall survival (OS) has not yet been shown [5,6,7].

Locoregional intraarterial treatments can be combined with other therapies like thermal ablation [8]. Several authors investigated the efficacy of TACE combined with thermal ablation for HCC, such as RFA [9,10,11,12] or MWA [13]. Their results of combination treatment of TACE and ablation were promising and generally showed a superiority to the monotherapy with TACE or ablation alone. The combination therapy of TACE and thermal ablation also showed promising results in treating liver metastases [14].

Only few studies have evaluated the combination treatment of bland TAE and ablation such as RFA and percutaneous ethanol injection (PEI) for HCC [15,16]. To our knowledge, no study has investigated the combination treatment of lipiodol-based TAE and MWA and compared it with MWA alone for HCC.

The purpose of this retrospective study was to investigate and compare the combination therapy of lipiodol-based TAE and MWA with the monotherapy MWA in treating HCC within the Milan criteria, mainly with regard to complications, oncological response, OS, and progression-free survival (PFS).

## 2. Materials and Methods

### 2.1. Patients’ Data

This comparative retrospective single-center study was approved by the institutional review board. A total of 112 patients (30 women and 82 males; mean age: 64.8 ± 10.1 years) with HCC within the Milan criteria [17], who were either treated by TAE followed by MWA as a combination therapy or by MWA as monotherapy with curative intent, were included.

HCC was diagnosed histologically and/or via contrast-enhanced MRI. 

The TAE-MWA group consisted of 49 patients (12 women and 37 males; mean age: 63.3 ± 9.6 years) with 55 tumors. Sixty-three patients (18 women and 45 males; mean age: 65.9 ± 10.5 years) with 67 tumors were enrolled in the MWA group. Both groups were homogenous with no significant differences in the baseline characteristics. The baseline characteristics of the patients and the tumors are shown in Table 1.

The inclusion criteria were (i) patients aged ≥18 years; (ii) single HCC lesion measuring ≤5 cm or up to three HCC lesions, each measuring ≤3 cm; and (iii) sufficient coagulation status. The exclusion criteria were (i) extrahepatic HCC manifestation; (ii) vascular invasion; and (iii) poor liver function (Child–Pugh class C).

The included patients’ cases were investigated with regard to age, sex, liver cirrhosis, and Child–Pugh class, location and number of tumors, preablation longest axial diameter of the tumor, complications, complete ablation, local tumor progression (LTP), intrahepatic distant recurrence (IDR), OS, and PFS.

### 2.2. TAE

Informed consent was obtained from all patients before TAE, which was performed according to our institutional protocol [18]. The last available contrast-enhanced cross-sectional imaging had been well evaluated in terms of the tumor location. Firstly, under sterile conditions, the femoral artery was punctured under local anesthesia using a needle, and a 5F-sheath was inserted via the Seldinger technique. Secondly, the coeliac trunk and superior mesenteric artery were catheterized using a 4F catheter.

An indirect portography was made to evaluate the portal venous system and exclude a portal vein thrombosis. Afterward, the catheter was placed in the common hepatic artery and then the right or left hepatic artery was selectively catheterized, depending on the location of the lesion. A 2.4F microcatheter was used to super-selectively catheterize the tumor-feeding arterial branches. When the target artery was reached, up to 10 mL of lipiodol (Guerbet, Villepinte, France) was carefully injected under fluoroscopy for the embolization of the tumor until blood stasis was reached. The patients in the TAE-MWA group received their MWA at least 24 h after TAE.

### 2.3. MWA

MWA was carried out under CT-guidance in all cases as previously described [19,20]. Before each MWA session, informed consent was obtained. All MWA therapies were performed under sedation. Monitoring of the vital signs was continuously performed during ablation. 

First, an unenhanced CT scan was performed to plan the procedure. After that, the patient was sterile-covered, and the puncture site was disinfected. After injecting the local anesthesia, the microwave antenna was placed in the HCC lesion. Following, the ablation was started, and the power was gradually increased. After completing the MWA, the application system was removed while sealing the needle track to avoid tumor seeding. The patients were discharged on the same day of treatment if no complications that required monitoring or therapy arose. Residual lesions were treated via one additional MWA session within four weeks after the initial MWA.

### 2.4. Follow-Up

In the TAE-MWA group, the patients received an unenhanced CT-scan within 24 h after the TAE to evaluate the lipiodol uptake and its distribution.

Contrast-enhanced MRI was used during the follow-up to assess tumor response after MWA in both groups. To analyze the ablation area, a contrast-enhanced MRI-scan was performed 24 h after the MWA session.

### 2.5. Definitions

Complications requiring additional treatments or longer hospitalization were considered as major complications [21].

A complete ablation was reached when the target HCC lesion was completely covered by the ablation area with a safety margin of at least 5 mm in the first contrast-enhanced MRI. The IDR was defined as the occurrence of new intrahepatic HCC lesions distant from the ablation area. The occurrence of a new HCC lesion along the ablation area during the follow-up was considered LTP [22]. The OS was calculated starting at the date of the tumor diagnosis as well as from the date of MWA until the date of the last follow-up or death. PFS was calculated from the date of the MWA until the date of LTP, IDR, or death.

### 2.6. Statistics

Continuous variables are presented as mean ± standard deviation and categorical variables as frequencies and percentages. Comparisons for categorical variables were completed using the Chi-square test or Fisher’s exact test, and for continuous variables, the Wilcoxon–Mann–Whitney U test was used.

The Kaplan–Meier estimator was used to calculate survival rates, including OS and PFS. Comparing survival between patients with monotherapy and combination therapy was performed with a log-rank test.

A two-sided *p*-value of ≤ 0.05 was considered statistically significant. Statistical analysis was performed with IBM SPSS Statistics, Version 29.0.

## 3. Results

### 3.1. Preablation Tumor Measurements

The mean diameter of the HCC lesions before ablation was 1.9 ± 0.7 cm in the TAE-MWA group and 2 ± 0.8 cm in the MWA group (*p* = 0.4). 

### 3.2. Complications

There were no procedure-related deaths or major complications in either group. Some patients complained about pain and/or nausea. These were treated symptomatically with commonly used medications.

### 3.3. Complete Ablation

Complete ablation was achieved in all lesions after the first MWA in the TAE-MWA group and in 97% (65/67) of the lesions in the MWA group (*p* = 0.5).

### 3.4. Oncological Response

**Figure 1:**
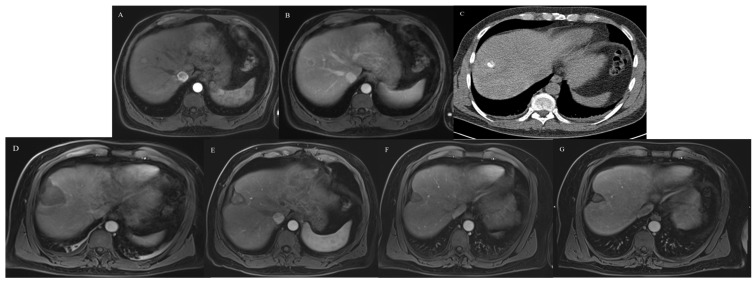
49-year-old male patient with HCC lesion in liver segment VIII treated by one session of lipiodol-based TAE, followed by CT-guided MWA. (**A**,**B**) Contrast-enhanced MRI scan shows typical pattern of HCC. (**C**) Unenhanced CT scan 24 h after TAE shows a compact accumulation of lipiodol within the tumor. (**D**) Contrast-enhanced MRI scan 24 h after MWA. (**E**) Contrast-enhanced MRI scan at 9-month follow-up. (**F**) Contrast-enhanced MRI scan at 2-year follow-up. (**G**) Contrast-enhanced MRI scan at 4-year follow-up shows only a small residue of ablation area without occurring of LTP.

**Figure 2:**
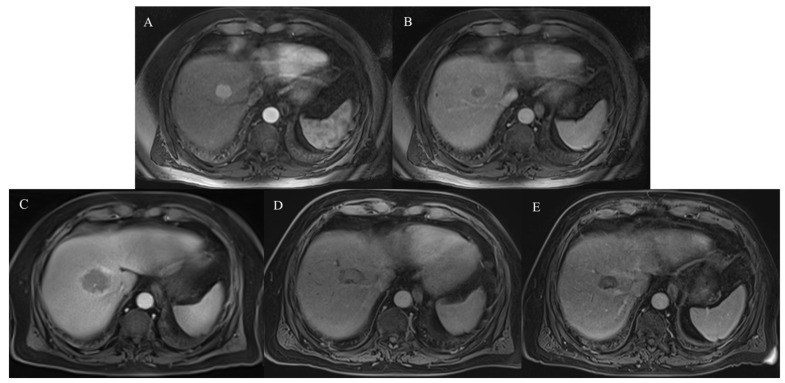
MWA monotherapy of histologically diagnosed HCC lesion in liver segment VIII in 80-year-old male patient. (**A**,**B**) Contrast-enhanced MRI scan shows the lesion prior to MWA. (**C**) Contrast-enhanced MRI scan 24 h after MWA. (**D**) Contrast-enhanced MRI scan at 6-month follow-up. (**E**) Contrast-enhanced MRI scan at 1-year follow-up. During the follow-up, the ablation area was shrinking without LTP.

The LTP rate was 5.5% (3/55) in the MWA-TAE group and 7.5% (5/67) in the MWA group (*p* = 0.73). The rate of IDR was 42.9% (21/49) in the MWA-TAE group and 52.4% (33/63) in the MWA group (*p* = 0.42). Patients’ cases are shown in Figure 1 and Figure 2.

### 3.5. Overall Survival

**Figure 3:**
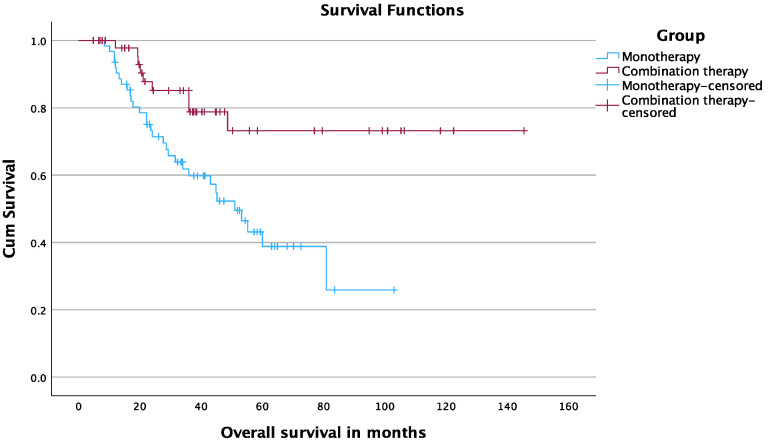
OS starting at date of diagnosis.

The 12-, 24-, and 36-month OS rates starting at the date of tumor diagnosis were 97.7%, 85.1%, and 78.8% in the TAE-MWA group and 91.9%, 71.4%, and 59.8% in the MWA group, respectively (*p* = 0.004) (Figure 3).

**Figure 4:**
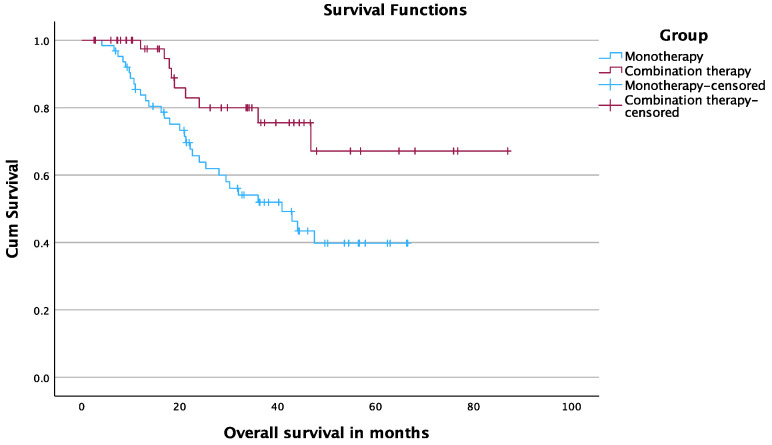
OS starting at date of MWA.

The 12-, 24-, and 36-month OS rates starting at the date of MWA were 97.4%, 80%, and 75.5% in the TAE-MWA group and 83.7%, 63.8%, and 51.9% in the MWA group, respectively (*p* = 0.009) (Figure 4).

### 3.6. Progression-Free Survival

**Figure 5:**
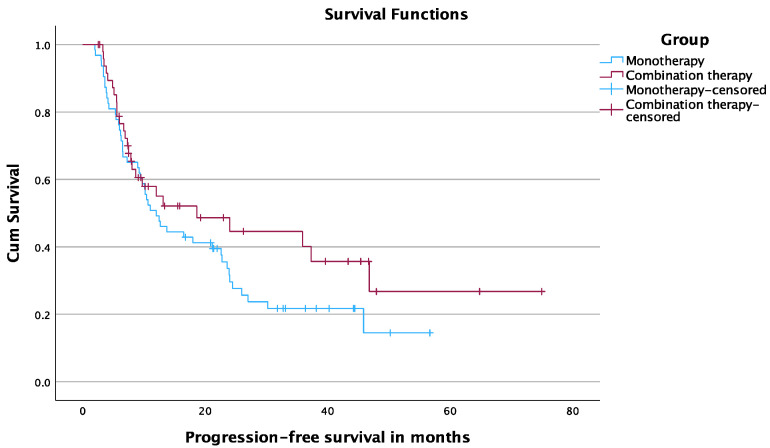
PFS.

The 6-, 12-, and 24-month PFS rates were 76.5%, 55%, and 44.6% in the TAE-MWA group and 74.6%, 49.2%, and 29.6% in the MWA group, respectively. The median PFS time was 18.6 months (95% CI, 0.64–36.5) in the TAE-MWA group and 12 months (95% CI, 8.05–15.9) in the MWA group (*p* = 0.18) Figure 5.

## 4. Discussion

Liver cancer remains a serious health issue that affects many patients and is one of the most common cancer-related deaths globally [23]. Treatment options for HCC are quite heterogeneous, varying from liver transplantation, surgical resection, and ablation to transarterial approaches, for instance [1].

There are many available embolization materials, including polyvinyl alcohol particles (PVA), lipiodol, or a gelatin sponge [8]. 

This retrospective study evaluated the combination therapy of lipiodol-based TAE and MWA in comparison to a monotherapy MWA and investigated which treatment protocol is more suitable for treating HCC within the Milan criteria. 

We found that the combination therapy of lipiodol-based TAE followed by MWA was superior to MWA alone in treating HCC within the Milan criteria. The patients in the TAE-MWA group had significantly better OS and non-significant better PFS, as well as local tumor control. Moreover, the initial complete ablation rate was in favor of the TAE-MWA group. We believe that the combination of TAE and MWA had a synergetic effect in treating HCC patients. The better local tumor control in the combination group may be the reason for the significantly better OS compared to the monotherapy group.

Bland TAE leads to ischemia and necrosis of the tumor by cutting off its blood supply [24]. The benefit of lipiodol-based TAE is not only the embolization itself and its consequences, but also to mark the tumor before thermal ablation [8]. As the ablation treatment is performed under the guidance of unenhanced CT, it is helpful to visualize the lesion using the hyperdense appearance of lipiodol prior to local ablation to accurately puncture the lesion and reduce the number of repositions of the MWA antenna.

Despite the safety of both treatment protocols in this study (with no reported major complications), it should not be forgotten that lipiodol-based embolization treatments can be associated with some very rare but serious complications, such as cerebral lipiodol embolism (CLE) [25,26,27]. These complications should also be considered before performing lipiodol-based treatments such as TACE or TAE, and patients should be well informed about them before any procedure.

In their prospective randomized trial, Peng et al. compared the combination therapy of TACE and RFA with RFA alone for HCC [11]. They showed better OS and recurrence-free survival (RFS) with the combination therapy. The 1- and 3-year OS rates were 92.6% and 66.6% in the TACE -RFA group and 85.3% and 59% in the RFA group, respectively. Our 1-, and 3-year OS rates starting at date of tumor diagnosis were 97.7% and 78.8% in the TAE-MWA group and 91.9%, and 59.8% in the MWA group, respectively. 

Maluccio et al. compared the combination therapy of bland TAE followed by RFA or PEI with the surgical resection of single HCC up to 7 cm in their retrospective study [15]. The RFS was better in the surgical group but the OS was similar in both groups. The reported 1-year OS rate was at 97% and the 3-year OS rate was at 77% in the combination therapy group. These survival rates are comparable to the OS rates in our TAE-MWA group. Song et al. investigated the combination therapy of RFA after TACE and compared it with TACE alone or RFA alone as a treatment for early HCC [10]. The rate of local recurrence was significantly lower in the combination treatment compared to TACE alone or RFA alone. In our study, the combination therapy also provided better local tumor control than the monotherapy. We reported rates of LTP at 5.5% in the MWA-TAE group and 7.5% in the MWA group. The rates of IDR were 42.9% in the MWA-TAE group and 52.4% in the MWA group, respectively. In a randomized study by Zaitoun et al., the combination therapy of TACE and MWA was compared with TACE alone or MWA alone in treating HCC >3–< 5 cm [13]. They found that the combination therapy was superior to TACE or MWA alone, and it provided significantly higher OS. In our current study, the OS was also significantly better in the combination therapy group compared to MWA alone.

Facciorusso et al. compared bland TAE with TACE for patients with HCC in a metanalysis of randomized trials [6]. They showed that the toxicity of TACE was significantly higher than that of TAE. They also reported that TACE is not superior to TAE when it comes to OS. In our opinion, if TACE before a thermal ablation (like MWA) provides the same or similar effects and benefits without significant differences in comparison to TAE, then it should be recommended to perform TAE combined with thermal ablation.

This study has several limitations. Firstly, the retrospective study design and its potential selection biases. Secondly, this is a single-center study with a relatively limited number of patients. Finally, though both groups were similar and lacked significant baseline differences, randomization of the patients is required. A prospective randomized study design would be more appropriate for this research question.

## 5. Conclusions

Both protocols were effective and safe as treatments for HCC within the Milan criteria, whether treating it via combination therapy or merely via monotherapy. The patients in the TAE-MWA group had significantly better OS, non-significant longer PFS, and better local tumor control than the patients in the MWA group.

## Figures and Tables

**Table 1:** Baseline characteristics of patients and tumors.

Characteristic	TAE + MWA	MWA	*p*-Value
No. of patients	49	63	
Mean age	63.3 ± 9.6 years	65.9 ± 10.5 years	0.136
Sex			
Women (%)	12 (24.5)	18 (28.6)	0.788
Male (%)	37 (75.5)	45 (71.4)	
Cirrhosis (%)	43 (88%)	58 (92%)	0.53
Child–Pugh class A (%)	35 (81)	40 (69)	0.158
Child–Pugh class B (%)	8 (19)	18 (31)	
No. of tumors	55	67	
≤2 cm (%)	32 (58.2)	35 (52.2)	
2–3 cm (%)	18 (32.7)	23 (34.3)	
≥3 cm (%)	5 (9.1)	9 (13.4)	
Solitary tumor (%)	43 (87.8)	59 (93.7)	0.329
≥two tumors (%)	6 (12.2)	4 (6.3)	
Location of tumor			
Right lobe (%)	35 (63.6)	42 (62.7)	
Left lobe (%)	19 (34.5)	21 (31.3)	
Both lobes (%)	1 (1.8)	3 (4.5)	
Caudate lobe (%)	0	1 (1.5)	

TAE, transarterial embolization; MWA, microwave ablation; No., number; W, women; M, men.

## Data Availability

The data may be requested from the corresponding author. All requests will be checked according to privacy and possible ethical restrictions.
